# Loss of Microtubule-to-Actin Linkage Disrupts Cortical Development

**DOI:** 10.1371/journal.pbio.1001175

**Published:** 2011-10-18

**Authors:** Richard Robinson

**Affiliations:** Freelance Science Writer, Sherborn, Massachusetts, United States of America

**Figure:**
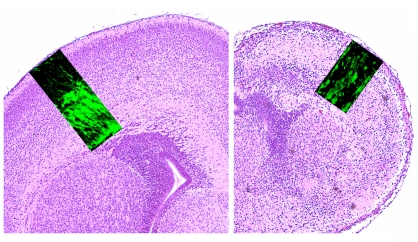
Histological sections from normal and Lis1-Nde1-deficient neonatal brains, formed during embryogenesis from radial glial cells labeled with green fluorescent protein.

Radial glial cells play a crucial role in the development of the brain; indeed, there would be no brain without them. They divide to give birth to new neurons during the formation of the central nervous system, and, stretching from the inner ventricles to the brain's outer surface, they also provide the scaffold along which the neurons migrate to their final locations.

Lissencephaly (“smooth brain”) is a developmental brain disorder caused by defects in both of these essential processes, neurogenesis and neuronal migration. One genetic cause of lissencephaly is loss of a protein called LIS1, which links to the microtubules and microtubule-based motors of the mitotic apparatus. Another cause is loss of NDE1, which binds to LIS1, but the details of how the loss of either causes lissencephaly is unclear. In this issue of *PLoS Biology*, Ashley Pawlisz and Yuanyi Feng show that Nde1 (the mouse version of NDE1) binds to dystrophin (a cytoskeleton protein) in order to build a multi-protein complex that links the extracellular matrix, actin cytoskeleton, and microtubules, stabilizing the radial glial cell membrane and facilitating neuronal migration.

The authors knew that the gene for Nde1 was expressed in radial glial cells. By carefully fixing cells to protect the plasma membrane, they found that a fraction of it localized to the cell surface. They used Nde1 as bait to find its binding partners (in addition to Lis1), and discovered that the protein bound to both dystrophin and utrophin. Dystrophin is an enormous protein that connects the actin cytoskeleton to the extracellular matrix. This is especially important for maintaining the structural integrity of muscle cells against the constant shear forces set up by their contraction. As such, loss of dystrophin causes Duchenne muscular dystrophy. Utrophin plays a similar role.

To bind to dystrophin (or utrophin), Nde1 used a site distinct from its binding site for Lis1, suggesting it may bind both simultaneously, and link them together. Loss of the dystrophin/utrophin binding site is known to be one cause of lissencephaly. When the authors depleted Nde1 in mice, dystrophin (but not utrophin), as well as an associated protein called dystroglycan, was largely lost from the membrane, and the radial glial cells were severely deformed, indicating the importance of the multi-protein complex for proper cell morphology. Deformed cells had reduced cell–cell adhesion, and failed to self-renew as normal cells do. The basement membrane of the cortex, a thin sheet of connective tissue at the outer limit of the brain to which radial glial cells attach, became disrupted, and many radial glial cells detached from the membrane.

Recall that newly born neurons must migrate along the long, thin radial glial cell for brain development. The loss of normal radial glial cell structure was accompanied by loss of normal neuronal migration, with too few neurons in some areas, and too many in others, a characteristic of one form of lissencephaly. Mice carrying these mutations died shortly after birth.

The authors conclude that the linkage between microtubules, actin, and the extracellular matrix provided by Nde1 and Lis1 furnishes the structural integrity needed by radial glial cells to both undergo normal division, and to facilitate the migration of neurons along their length. These results are likely to provide insights into not only the rare disorders of human brain development, but also the complex interactions required for development of the normal cortex.

**Pawlisz AS, Feng Y (2011) Three-Dimensional Regulation of Radial Glial Functions by Lis1-Nde1 and Dystrophin Glycoprotein Complexes. doi:10.1371/journal.pbio.1001172**

